# Short-chain fatty acid attenuates intestinal inflammation by regulation of gut microbial composition in antibiotic-associated diarrhea

**DOI:** 10.1515/biol-2022-0931

**Published:** 2025-03-13

**Authors:** Li Lin, Lihong Han, Cuihong Gu, Lihong Wang, Zhihua Zhang

**Affiliations:** Department of Hematology, The Affiliated Hospital of Chengde Medical College, Chengde, Hebei 067000, China; Department of Geratology, The Affiliated Hospital of Chengde Medical College, Chengde, Hebei 067000, China; Department of Hematology, The Affiliated Hospital of Chengde Medical College, No. 36, Nanyingzi Street, Chengde, Hebei 067000, China

**Keywords:** hematologic malignancies, short-chain fatty acid, antibiotic-associated diarrhea, gut microbiota, intestinal inflammation

## Abstract

To investigate fecal short-chain fatty acid (SCFA) levels in hematological malignancies (HMs) patient with antibiotic-associated diarrhea (AAD), and explore the impacts of SCFAs on intestinal inflammation and gut microbiota in rats with AAD. Fecal SCFA concentrations were determined by high-performance liquid chromatography. Histologic examination was conducted by hematoxylin–eosin and alcian blue–Periodic acid–Schiff. Interleukin (IL)-10 and IL-18 mRNAs were assessed by quantitative real-time polymerase chain reaction. Claudin3 (CLDN3), Zona Occludens 1 (ZO-1), and plasmalemma vesicle-associated protein (PLVAP) proteins were evaluated by immunofluorescence and western blot. Gut microbiota was assessed by 16S rRNA sequencing. SCFAs are decreased in fecal samples of HM patients with AAD. AAD incidence is correlated with serum albumin level and type/duration of antibiotics administered. SCFAs attenuate colon shortening and intestinal pathology, and reinstate functionality of intestinal barrier by upregulating CLDN3/ZO-1 and downregulating PLVAP. Control (ctrl) group harbors an increased abundance of *Lactobacillus*, AAD group exhibits an enrichment of *Enterorhabdus*, AAD + low (L)-SCFAs group displays a predominance of *Turicibacter*, and AAD + high (H)-SCFAs group exerts an enrichment of *Clostridium* IV. Altogether, SCFAs alleviate colonic inflammation by regulating gut microbial composition, and provide insight into enhancing intestinal SCFAs content to alleviate AAD-induced symptoms in HM patients by modifying dietary structure.

## Graphical abstract

**Figure:**
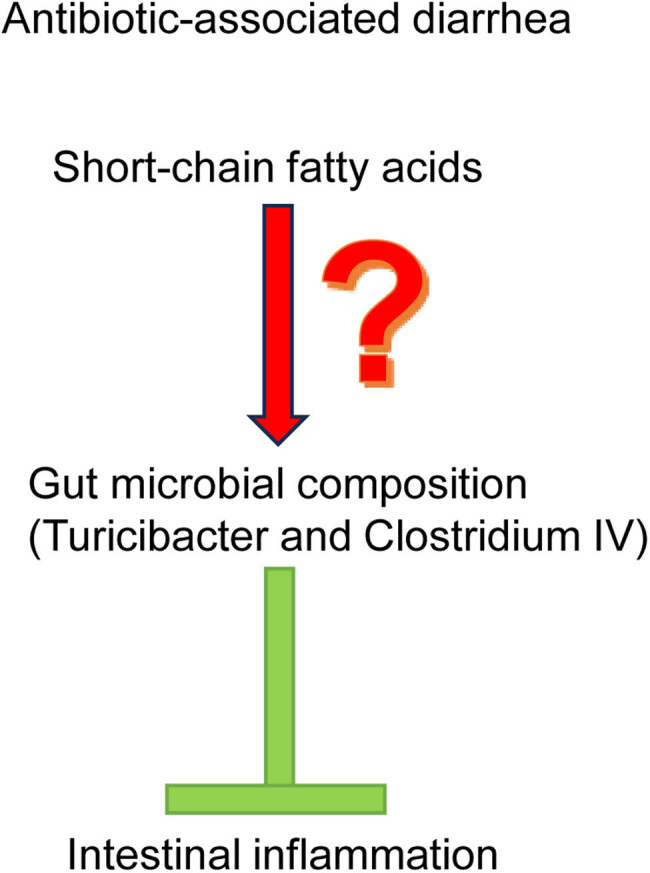

## 1 Introduction

Hematologic malignancies (HMs) encompass a group of malignant transformations which arise from cells located in the primary or secondary lymphoid organs. The three primary types of HMs are leukemia, lymphoma, and multiple myeloma [1]. With the current application of novel cancer treatment modalities, including immunotherapy [2], targeted therapy [3], and particularly small molecule targeted agents [4], the overall survival of patients with HMs has been substantially prolonged. However, due to neutropenia or defective neutrophil functions, impaired T- and B-lymphocyte functions, and defects of vascular endothelial barrier functions, the infection incidence remains 20.8–24.1% in patients with HMs [5].

A multitude of broad-spectrum antibiotics have revolutionized the treatment of infectious diseases. However, the widespread and irrational utilization of antibiotics caused numerous problems; one of the most compelling issues is antibiotic-associated diarrhea (AAD) [6]. As acknowledged, the occurrence of AAD is linked with the dysbiosis of gut microbiota and their metabolites [7].

Gut microbiota, a diverse community of trillions of microbes, are crucial in the development and physiology of the host [8]. Probiotics administration is a reasonable therapy to rescue gut microbiota [9]. As well known, the intestine health relies on the metabolic compound such as short-chain fatty acids (SCFAs) [10]. SCFA receptors and gut microbiota act as therapeutic targets in immune diseases [11], and SCFAs serve as a possible treatment option for autoimmune diseases [12]. In addition, natural compounds and/or their derivatives enhance the intestinal mucosal barrier function and reduce intestinal mucosal damage in AAD mice by increasing the abundance of SCFAs-producing bacteria [13]. Moreover, in 2024, a new published study reports that milk-derived *Lactobacillus* with high production of SCFAs relieves AAD in mice [14]. However, the effects of intestinal supplementation with SCFAs on AAD rats are less studied.

Herein, the clinical profile and fecal SCFAs content from patients with HMs who were treated with antibiotics were evaluated. Meanwhile, by establishing a rat model of AAD, the effects of intestinal supplementation with SCFAs on body weight, cecum index, colon morphology, barrier function, and gut microbiota were investigated.

## 2 Materials and methods

### 2.1 Patients

Patients with HMs (*n* = 113) who received antibiotic treatment at the affiliated hospital of Chengde Medical College in China between Dec 2020 and Dec 2023 were enrolled in the present study. In total, there were 92 patients without AAD (non-AAD group) and 21 patients with AAD (AAD group). For AAD cohort, fecal SCFAs were analyzed by high-performance liquid chromatography (HPLC) before and after AAD.


**Informed consent:** Informed consent has been obtained from all individuals included in this study.
**Ethical approval:** The research related to human use has been complied with all the relevant national regulations, institutional policies and in accordance with the tenets of the Helsinki Declaration, and has been approved by the Ethical Committee of the Affiliated Hospital of Chengde Medical College.

### 2.2 Experimental design in animals

Sprague Dawley rats (250  ±  30 g, Beijing Weitong Lihua Experimental Animal Technology Co., Ltd) were housed under a controlled condition: at temperature of 22 ± 0.5°C, a humidity of 50 ± 5%, light: dark cycles of 12:12 h, and free to standard laboratory pellets and water. Animal experiments were performed in strict accordance with the Guidelines for the Care and Use of Laboratory Animals and the Chinese Legislation Laboratory Animals. The present study was approved by the ethical committee of The Affiliated Hospital of Chengde Medical College. Each effort was made to maximize the well-being of rats, and minimize their suffering and the number of animals used.

After acclimatizing the animals, 24 rats were randomly distributed into four groups (*n* = 6/group), including control group (ctrl), model group (AAD), low dosage SCFAs treatment group (AAD + L-SCFAs), and high dosage SCFAs treatment group (AAD + H-SCFAs). Rats in ctrl group were subjected to gavage with 0.9% saline (0.01 L/kg) for 14 consecutive days. Rats in AAD, AAD + L-SCFAs, and AAD + H-SCFAs groups were subjected to gavage with 0.9% saline (0.01 L/kg), 0.1 g/kg, and 0.15 g/kg SCFAs, respectively, for the first 7 days, while lincomycin hydrochloride (3 g/kg) was administered by gavage once a day for the second 7 days to obtain AAD model (soft feces with normal shape, watery stool, and mucous stool were regarded as diarrhea) [15]. During the treatment period, body weight of each rat was measured daily. Rats were killed on Day 14. Colon tissues were collected for histological assessment and DNA extraction. The cecum was rinsed, weighed, and frozen in liquid nitrogen. The cecum index was calculated as follows: cecum index = cecum weight (mg)/body weight (g).


**Ethical approval:** The research related to animal use has been complied with all the relevant national regulations and institutional policies for the care and use of animals, and has been approved by the Ethical Committee of the Affiliated Hospital of Chengde Medical College.

### 2.3 Histopathological analysis of colon

The distal colon was flushed with ice-cold PBS, fixed in 4% paraformaldehyde overnight, embedded in paraffin, sliced into 5 μm sections, and incubated for 30 min at 60°C. Sections (5 μm) were subjected to hematoxylin–eosin (HE) staining and alcian blue–Periodic acid–Schiff (AB/PAS) staining, respectively. Thereafter, sections were dewaxed by xylene I (15 min) and xylene II (15 min). Next, sections were hydrated by absolute ethanol (5 min), and 90, 80, 70% ethanol (3 min/each).

As for HE staining, sections were immersed in 10% hematoxylin (5 min), differentiated by 75% ethanol hydrochloride (5 s), and stained by 0.5% eosin (3 min). As for AB/PAS staining, sections were immersed in alcian blue (10 min), oxidized by Periodic acid (5 min), immersed in Schiff reagent (10 min) and 10% hematoxylin (2 min), differentiated by 75% ethanol hydrochloride (5 s), and stained by Scott blue solution (2 min).

Afterward, sections were dehydrated by alcohol gradient 70, 80, 90% (1 min/each) and absolute ethanol (4 min). Subsequently, sections were permeabilized by xylene I (10 min) and xylene II (10 min). At last, images were observed by a microscope (Olympus). Image Pro-Plus 6.0 was applied for data analysis.

### 2.4 Quantitative real-time polymerase chain reaction (qRT-PCR)

Total RNA was extracted from colonic tissues by Trizol reagent (Thermo Fisher Scientific), and reverse transcribed into cDNA by PrimeScript qRT-PCR Reagent kit (Takara). Relative mRNA levels were estimated using qRT-PCR assays with SYBR Green qPCR Master Mix and StepOnePlus (Applied Biosystems). The expression levels of interleukin (IL)-10 and IL-18 were analyzed by 2^−ΔΔCt^ method, and normalized to the internal control GAPDH. The primer sequences are listed in Table 1.

**Table 1:** Primer sequences

Genes	Accession number	Forward (5′−3′)	Reverse (5′−3′)	Amplicon length (bp)
IL-10	NM_012854.2	CGGGGTGACAATAACTGCACCC	CTGTCAGCAGTATGTTGTCCAGC	130
IL-18	NM_019165.2	ACCCGCCTGTGTTCGAGGACATG	TGTTTTTACAGGAGAGGGTAGAC	159
GAPDH	NM_017008.4	GTCTCCTGTGACTTCAACAGCA	ACCACCCTGTTGCTGTAGCCAT	131

### 2.5 Western blot

Colon tissues were lysed by RIPA buffer containing protease inhibitor cocktail. Protein samples were subjected to SDS-PAGE (12%, Bis-Tris Midi Gel, Invitrogen) for electrophoresis to PVDF membranes. After blocking with BSA (5%) for 1 h at room temperature, PVDF membranes were incubated with primary antibodies against plasmalemma vesicle-associated protein (PLVAP; 1:1,000) and Claudin3 (CLDN3; 1:1,000) overnight at 4°C; after being washed by TBST, PVDF membranes were incubated with secondary antibodies for 1 h at room temperature. After being rinsed thoroughly, HRP signals were detected by ECL western blotting substrate (Pierce). The intensities of protein bands were analyzed by ImageJ. Proteins were normalized to the internal control GAPDH.

### 2.6 Immunofluorescence

Sections (5 μm) were deparaffinized, rehydrated, and washed by PBS. Afterward, sections were blocked with 2% BSA for 30 min at room temperature, and then incubated with the primary antibodies against Zona Occludens 1 (ZO-1; 1:200) and PLVAP (1:200) overnight at 4°C. Thereafter, sections were incubated with fluorescence labeled anti-rabbit IgG secondary antibody for 1 h at room temperature, and co-incubated with DAPI for 4 min in the dark at room temperature. At last, images were observed by a microscope (Olympus). Image Pro-Plus 6.0 was applied for data analysis.

### 2.7 16S rRNA gene sequence analysis of intestinal flora in fecal samples

DNA of the intestinal contents was extracted by E.Z.N. A™ Mag-Bind Soil DNA Kit (OMEGA), quantified by Qubit dsDNA HS Assay Kit (ThermoFisher), and visualized by agarose gel electrophoresis. The V3–V4 region of 16S rDNA gene was amplified by PCR with the following primers: forward, 5′-CCTACGGGNGGCWGCAG-3′ and reverse, 5′-GACTACHVGGGTATCTAATCC-3′. Finally, the sequencing data were subjected to bio-informatics analysis to analyze the diversity and composition of gut microbiota in AAD rats.

The diversity and richness of the communities were compared by alpha diversity indices, including Chao index and Simpson index. The composition of the microbial community was explored by β-diversity. The fecal microbiota taxa with the greatest difference were analyzed by the LEfSe method. With the LDA score set at 4.0 as the screening standard, the microbes with high abundance were determined.

### 2.8 Statistical analysis

Statistical analysis was carried out by GraphPad Prism 8.0. Each experimentation was conducted more than three times. Difference in patients between two groups (before and after AAD) was analyzed by paired Student’s *t*-test. Differences in AAD rats among four groups (ctrl, AAD, AAD + L-SCFAs, and AAD + H-SCFAs) were analyzed by one-way analysis of variance analysis followed with Tukey’s test. Data are expressed as mean ± standard deviation. Statistical significance was defined as *P* < 0.05.

## 3 Results

### 3.1 Influential factors and fecal SCFAs content in HM patients with AAD

As shown in Table 2, compared to ctrl, HM patients who developed AAD exhibited a prolonged duration of antibiotic exposure, and a more frequent use of enzyme inhibitor antibiotic therapy. Furthermore, the albumin level during the consultation was correlated with the incidence of AAD. Nevertheless, compared to ctrl, there was no significant correlation between the incidence of AAD and variables such as gender, age, and primary disease.

**Table 2:** Basic characteristics of patients in AAD group and non-AAD group

	AAD	Non-AAD	*F*	*P*
Sex (*n*, %)			27.77	0.07
Female	10 (47.6)	65 (70.7)		
Male	11 (52.4)	27 (29.3)		
Age (*n*, %)			29.46	0.92
<60	10 (47.6)	45 (48.9)		
≥60	11 (52.4)	47 (51.1)		
Diagnosis (*n*, %)			25.90	0.31
Leukemia	15 (71.4)	76(82.6)		
Multiple myeloma	3 (14.3)	9 (9.8)		
Lymphoma	3 (14.3)	7 (7.6)		
Antibiotic time (day)	21.5 ± 11.2	14.0 ± 8.9	26.13	0.01
Antibiotic classes (*n*, %)			24.46	0.01
1	2 (9.5)	27 (29.3)		
2	4 (19.0)	28 (30.4)		
3	4 (19.0)	19 (20.7)		
4	4 (19.0)	9 (9.8)		
5	3 (14.3)	3 (3.3)		
6	2 (9.5)	4 (4.3)		
7	2 (9.5)	2 (2.2)		
Antibiotics (*n*, %)				
Cephalosporin	8 (38.1)	48 (52.2)	30.03	0.25
Quinolones	11 (52.4)	28 (30.4)	27.94	0.08
Glycopeptides	8 (38.1)	14 (15.2)	25.02	0.06
Carbapenems	7 (33.3)	17 (18.5)	26.28	0.20
Antifungal	17 (81.0)	59 (64.1)	34.45	0.11
Enzyme inhibition	16 (76.2)	42 (45.7)	33.17	0.01
Albumin levels on admission (g/L)	33.8 ± 6.0	36.9 ± 4.8	26.02	0.04

**Figure 1:**
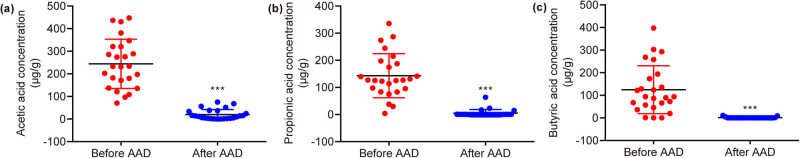
Expression profiles of fecal SCFAs between HM patients who developed AAD and those who did not. The changes of acetic acid (a), propionic acid (b), and butyric acid (c) in HM patients before and after AAD. *N* = 25. ***P < 0.001.

HPLC analysis revealed a notable reduction in fecal SCFAs in HM patients with AAD, including acetic acid (Figure 1a), propionic acid (Figure 1b), and butyrate acid (Figure 1c); remarkably, acetic acid exerted the most substantial decline.

### 3.2 SCFAs mitigated intestinal inflammation in rats with AAD

**Figure 2:**
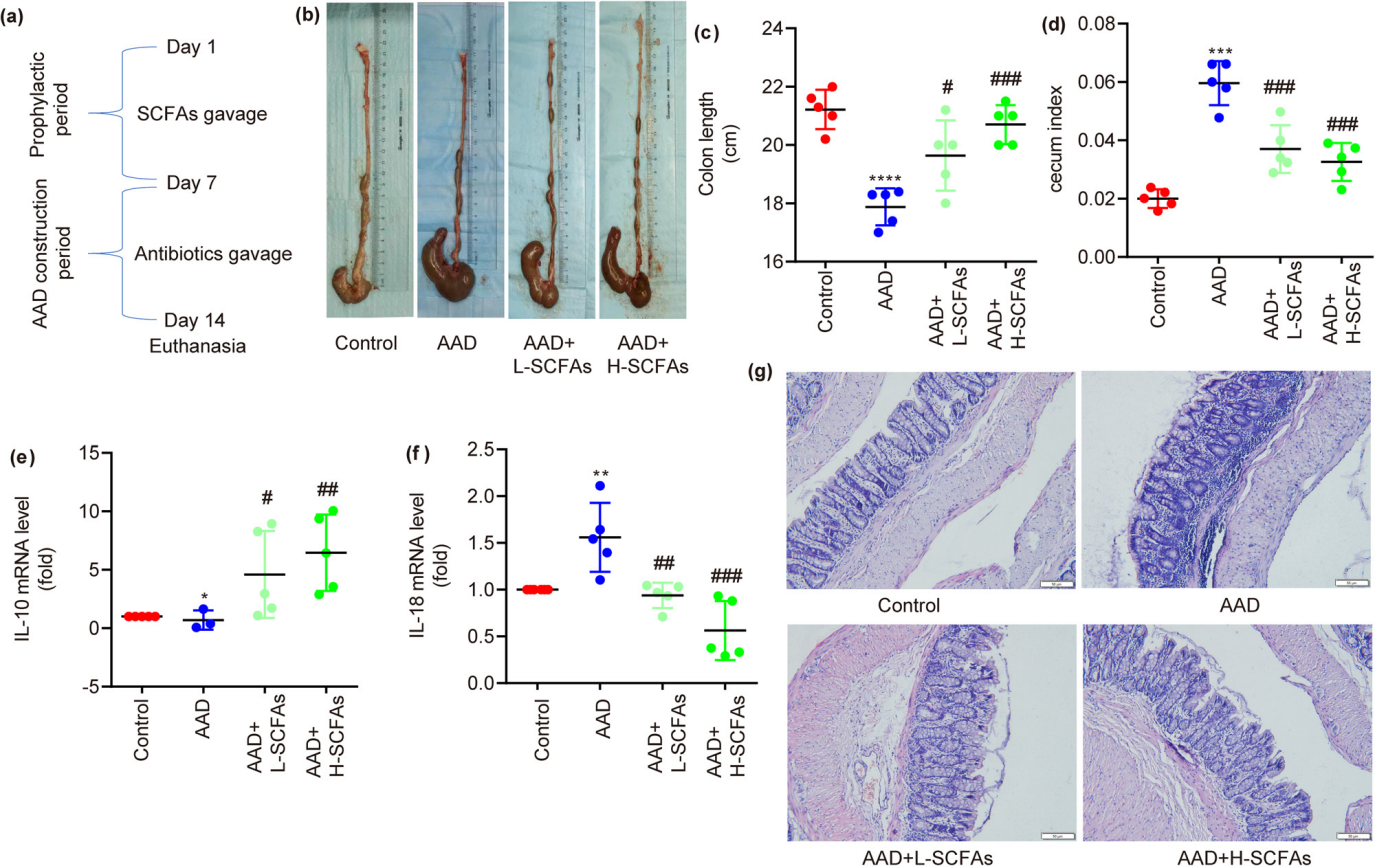
SCFAs attenuate lincomycin susceptibility and intestinal inflammation in rats. A timeline scheme for this experiment is presented (a). Colon length is presented (b) and (c). Cecum index is measured (d). mRNA levels of IL-10 and IL-18 are measured by qRT-PCR (e) and (f). HE staining of rat colonic tissue is shown (g). Scale bars = 20 μm; *N* = 5. *P < 0.05, **P < 0.01, ***P < 0.001, ****P < 0.0001.

A timeline scheme for this experiment is shown in Figure 2a. Compared to ctrl, colon length was shortened and cecal index was increased by AAD, which were reversed by SCFAs dose-dependently (Figure 2b–d). In comparison with ctrl, colonic mRNA expression level of IL-10 was decreased and colonic mRNA expression level of IL-18 was increased in rats with AAD, which were reversed by SCFAs dose-dependently (Figure 2e and f). Besides, HE staining showed that compared with ctrl, there was obvious inflammatory cell infiltration in rats with AAD, which was mitigated by SCFAs dose-dependently (Figure 2g).

Hence, the metabolic process of SCFAs leads to the production of acetic, propionic, and butyric acids, thus inhibiting inflammation.

### 3.3 SCFAs enhanced the integrity of the intestinal barrier in rats with AAD

**Figure 3:**
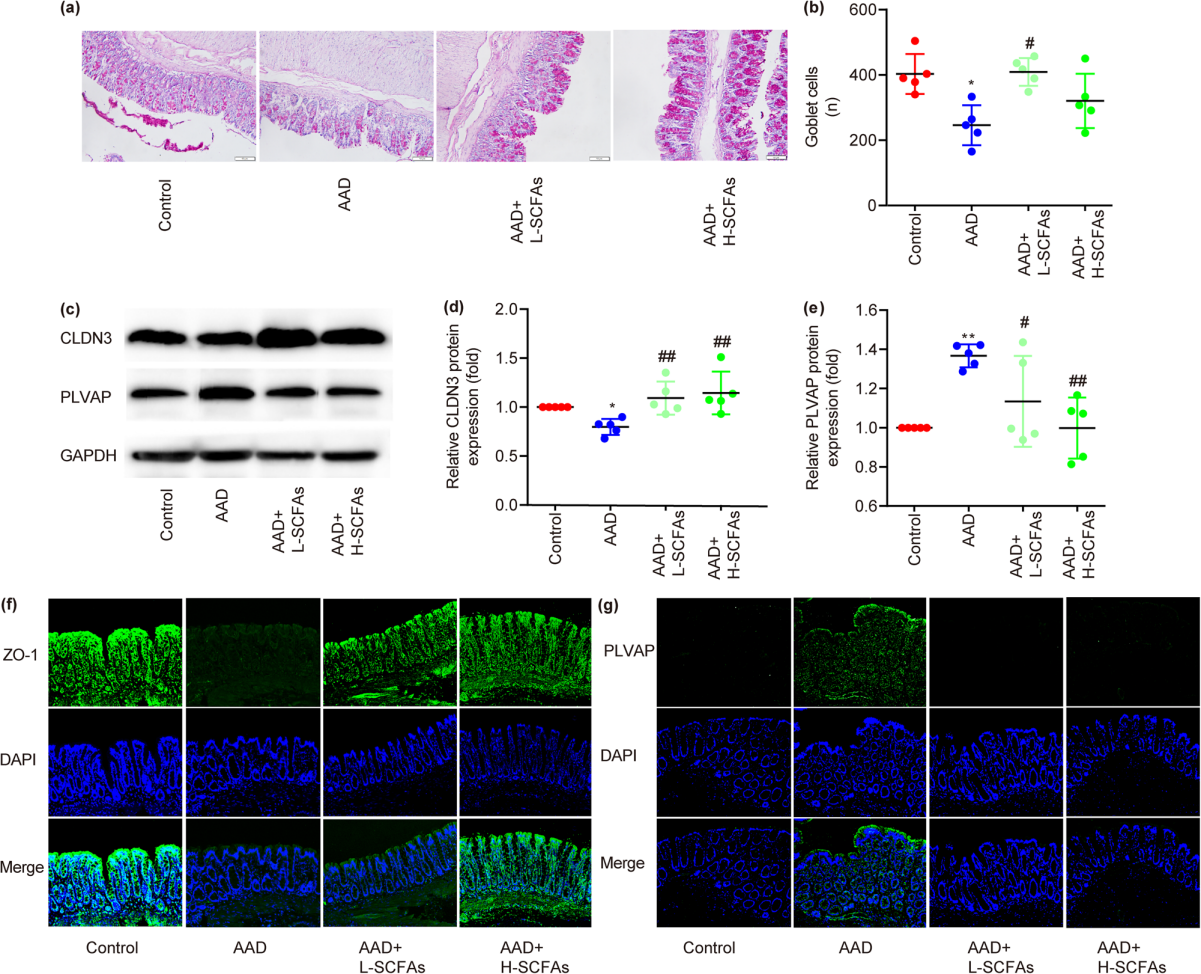
SCFAs enhance intestinal epithelial barrier function. The effects of SCFAs on mucous-secreting goblet cells are shown by AB/PAS staining (a) and (b). Protein expressions of CLDN3 and PLVAP are examined by western blot (c)–(e). Protein expressions of ZO-1 and PLVAP are examined by immunofluorescence microscopy (f) and (g). *N* = 5. *P < 0.05 vs control, **P < 0.05 vs AAD.

In addition, AB–PAS staining was conducted to investigate goblet cell hyperplasia in the colon. It demonstrated that compared with ctrl, goblet cell hyperplasia was decreased in rats with AAD, which was partially reversed by SCFAs (Figure 3a and b).

Subsequently, rat colonic tissues were collected for evaluating the integrity of the intestinal barrier by immunofluorescence staining and western blotting. Western blotting revealed that, compared to ctrl, CLDN3 protein expression was significantly decreased, while PLVAP protein expression was significantly increased in rats with AAD, which were partially attenuated by SCFAs in a dose-dependent manner (Figure 3c–e). Immunofluorescence staining showed that, compared to ctrl, ZO-1 protein expression was significantly decreased, while PLVAP protein expression was significantly increased in rats with AAD, which were partially attenuated by SCFAs (Figure 3f and g).

### 3.4 SCFAs induced changes in the composition and diversity of gut microbiota in rats with AAD

We applied 16S RNA to elucidate the effect of SCFAs on the diversity and composition of gut microbiota in rats with AAD.

**Figure 4:**
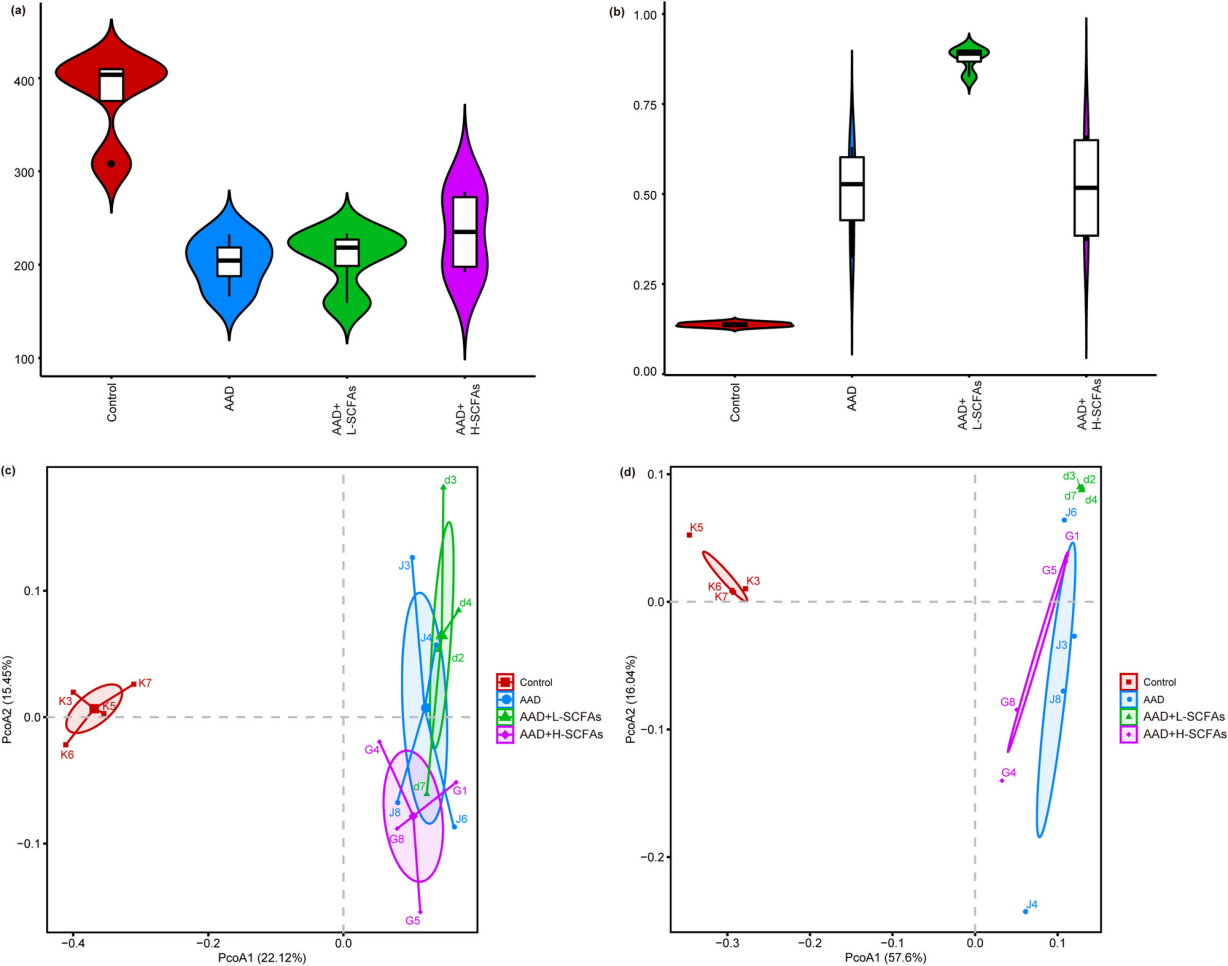
Effect of the intervention with SCFAs in gut microbiota composition. The alpha diversity of the gut microbiome, including Chao (a) and Simpson indices (b). Beta diversity analysis based on the unweighted (c) and weighted (d) Unifrac index. *N* = 5.

Compared to ctrl, the Chao and Simpson indexes were decreased by AAD; however, they were not reversed by SCFAs (Figure 4a and b). Weighted and unweighted UniFrac analyses demonstrated that compared to ctrl, the composition of the microbial community was significantly changed by AAD; however, it was not reversed by SCFAs (Figure 4c and d).

**Figure 5:**
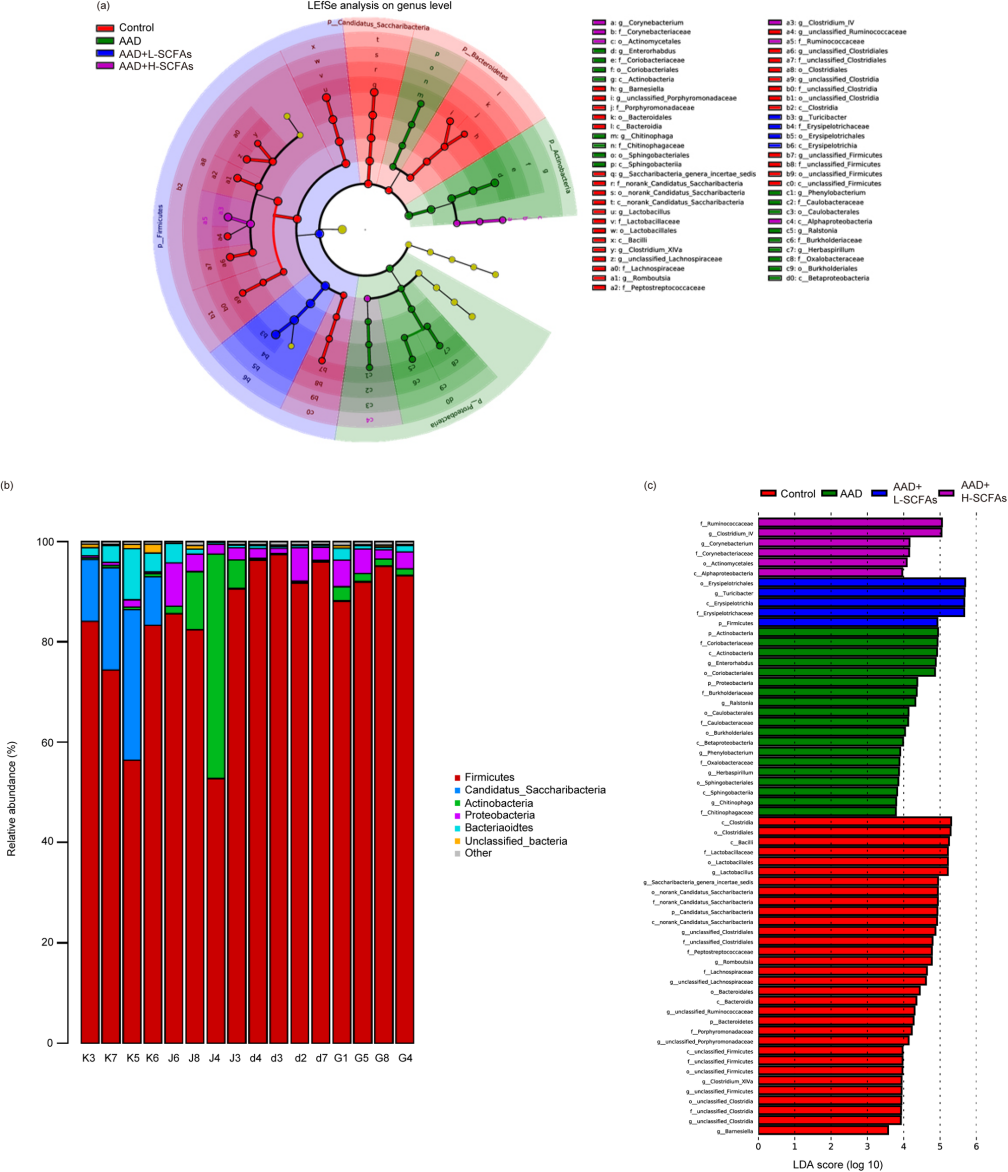
Effect of the intervention with SCFAs in gut microbiota diversity. SCFAs modulated gut microbiota at phylum levels and LEfSe analysis of the dominant biomarker taxa among the four groups. Evolutionary cladogram (a), phylum level (b), and LDA score distribution histogram (c).

In ctrl group, *Lactobacillus* had the highest abundance, AAD group was dominated by Enterobacteriaceae, while L-SCFAs group was abundant with genus *Turicibacter* of phylum Firmicutes, and H-SCFAs group was enriched with *Clostridium* IV (Figure 5a–c). In conclusion, SCFAs can alter the composition and diversity of gut microbiota in AAD rats.

## 4 Discussion

As one of the most common complications of antibiotic use, AAD has attracted more and more attention recently [6]. Consistent with a previous study [16], we also observed that in 112 patients with HMs who were administered with antibiotics, the incidence of AAD was 18.94%, which was influenced by multiple factors, e.g., the duration/type of antibiotic administered and the level of albumin during consultation. However, current analysis did not find a correlation between the prevalence of AAD and primary pathology, age, or gender. Moreover, by establishment of an AAD model in rats, SCFAs were discovered to attenuate clinical symptoms, improve intestinal barrier function, and alleviate intestinal inflammation by modulating gut microbiota.

As acknowledged, cytokines are major regulators of mucosal immunity and intestinal immune defense, changes in cytokines represent the intestinal immune function when combined with AAD [17]. Anti-inflammatory cytokine such as IL-10 restores the function of disrupted barriers; however, pro-inflammatory cytokines such as IL-18 increase intestinal epithelial tight junction permeability, and result in intestinal barrier dysfunction [18]. Interestingly, IL-10 expression can be upregulated by SCFAs and intestinal epithetical barrier can be enhanced by SCFAs [19,20]. Additionally, parthenolide increases IL-10 expression by improving the balance of Treg/Th17 in intestinal mucosa, which is mediated by increased production of microbiota-derived SCFAs [21]; moreover, by increasing the content of SCFAs, *Pulsatilla* decoction repairs the colonic mucosal barrier in ulcerative colitis mice by increasing IL-10 concentration [22]. Regarding IL-18, sanguinarine relives colitis by inactivating NLRP3-Caspase1/IL-1β pathway, which in turn upregulates IL-18 to exert proinflammatory effects [23]. Ketogenic diet protects intestinal barrier function by reducing the production of IL-18 [24]. In a recent study, Pu et al. proposed a dual role of IL-18, particularly in a colitis, wherein IL-18 exhibits both pro-inflammatory and anti-inflammatory effects, depending on the natures of the invading organisms [25]. In consistence with previous studies [19,20,24], our results demonstrated that SCFAs supplementation increased the expression of IL-10 and decreased the expression of IL-18. Hence, SCFAs may exert anti-inflammatory effects by regulating the expressions of cytokines.

The barrier function of epithelium is the first line of defense of the intestine. Intestinal epithelial cells maintain the integrity and tight junction of intestinal epithelium by promoting the protein expression such as ZO-1 [26], oral gavage of mulberry anthocyanins improved intestinal barrier function and restored the expressions of intestinal tight junction protein (ZO-1 and CLDN3) in mice with dextran sulfate sodium-induced colitis [27]. PLVAP is a primary factor influencing the permeability of endothelial cells, and regulates vascular permeability [28]. Herein, there were fewer mucus-secreting cuprocytes in AAD rats, which was reversed by SCFAs. Meanwhile, SCFA increased the expression of ZO-1/CLDN3 and improved mucosal barrier function, while decreased the expression of PLVAP and intestinal permeability in AAD rats, which are in line with previous literature [29,30].

In the current study, we found that L-SCFAs enriched *Turicibacter* abundance. As a genus, *Turicibacter* is linked to impaired glucose and lipid metabolism, displaying a negative correlation with random blood glucose levels in diabetic adipose rats [31]. Additionally, *Turicibacter* is associated with the presence of intestinal butyric acid, which enhances insulin secretion/sensitivity and exhibits notable anti-obese and anti-inflammatory properties [32]. Moreover, the decrease of *Turicibacter* is associated with inflammation [33], and an increase in consumption of cholesterol/fat in the diet [34]. The finding in the current study suggests that the anti-inflammatory effects of SCFAs may be related to interference with the gut microbiota associated with glycolipid metabolism. Besides, H-SCFAs enriched the abundance of *Clostridium* IV. *Clostridium* IV is specialized in the breakdown of fiber, diets enriched in different types of fibers, like inulin, guar gum, oligofructose, arabinoxylan, and resistant starch can induce the enrichment of *Clostridium* IV [35]. *Clostridium* IV is a bacterium that metabolizes various carbohydrates as the exclusive carbon and energy source. This metabolic process leads to the production of acetic and butyric acids, acetone, butanol, ethanol, CO_2_, and H_2_, thereby exerting anti-inflammatory effects.

However, there remain several limitations in the current study: (1) Intestinal microecology includes intestinal structure, intestinal microorganisms, and microbial metabolites; however, the current study focuses only on microbial metabolites. (2) The application of microbiota transplantation and other means are needed for further validation. (3) The exploration about the protective effect of SCFAs on the intestinal mucosa of AAD is at the preliminary stage and needs to be further studied. (4) The mechanisms by which SCFAs modulate the gut microbiota need to be clarified in the future work.

Collectively, SCFAs down-regulate the level of inflammatory factors by regulating intestinal flora, enhance the intestinal mucosal barrier function, and improve the diarrhea symptoms in AAD rats, thus providing a theoretical basis for the anti-inflammatory effect of increasing intestinal SCFAs concentration by adjusting the dietary structure.
